# Downregulation of Ras Association Domain Family Member 6 (RASSF6) Underlies the Treatment Resistance of Highly Metastatic Nasopharyngeal Carcinoma Cells

**DOI:** 10.1371/journal.pone.0100843

**Published:** 2014-07-16

**Authors:** Ying-Ying Liang, Ming-Yuan Chen, Yi-Jun Hua, Shi Chen, Li-Sheng Zheng, Xue Cao, Li-Xia Peng, Ping Xie, Bi-Jun Huang, Rui Sun, Lin Wang, Yan-Qun Xiang, Xiang Guo, Chao-Nan Qian

**Affiliations:** 1 State Key Laboratory of Oncology in South China and Collaborative Innovation Center for Cancer Medicine, Sun Yat-sen University Cancer Center, Guangzhou, China; 2 Department of Nasopharyngeal Carcinoma, Sun Yat-sen University Cancer Center, Guangzhou, China; 3 Department of Gastroesophageal surgery, The Sixth Affliated Hospital (Gastrointestinal and Anal Hospital), Sun Yat-sen University, GuangZhou, China; The University of Hong Kong, China

## Abstract

Radiation and cisplatin-based chemotherapy are major treatments for nasopharyngeal carcinoma (NPC). However, a major impediment for further improving the cure rate is the development of treatment resistance with an undetermined molecular mechanism in metastatic NPC cells. Our established, highly metastatic NPC cells have been reported to be more resistant to cisplatin chemotherapy. In the present study, we found that Ras association domain family member 6 (RASSF6) was downregulated in highly metastatic cells but upregulated in low metastatic cells in comparison to their parental cell line. Ectopic-expression of RASSF6 enhanced the sensitivity of highly metastatic NPC cells to cisplatin or radiation by enhancing apoptosis. RASSF6 depletion conversely reduced treatment sensitivity by decreasing the apoptosis rate. Over-expression of RASSF6 in highly metastatic NPC cells could enhance the phosphorylation of JNK when exposed to cisplatin or radiation treatment, while knocking down RASSF6 in low metastatic NPC cells could reduce the level of phospho-JNK when exposed to the same treatments. The activation of JNK signaling by RASSF6 and its subsequent sensitivity to apoptosis in NPC cells could be inhibited by applying the JNK inhibitor SP600125. In conclusion, the downregulation of RASSF6 in highly metastatic NPC cells contributed to their treatment resistance, and over-expression of RASSF6 conferred treatment sensitivity to highly metastatic NPC cells by activating JNK signaling. RASSF6 could be a valuable molecular marker for identifying sensitive metastatic NPC tumors during cisplatin treatment or radiotherapy.

## Introduction

Nasopharyngeal carcinoma (NPC) is a common malignancy in southern China and south-east Asia that has the highest metastasis rate among head and neck cancers [1], [2]. NPC is relatively sensitive to radiation therapy and chemotherapy. For those newly diagnosed with loco-regional advanced NPC, which accounts for approximately 70% of cases, concurrent chemo-radiotherapy is the standard for care, while cisplatin-base chemotherapy is the first-line chemotherapy regimen. However, distant metastasis will eventually occur in approximately 20% of NPC patients after the standard treatment [3]–[5]. Metastatic lesions of this particular malignancy are more frequently resistant to further chemotherapy or radiation therapy due to undetermined mechanisms. As a consequence, distant metastasis is currently the main reason for treatment failure in NPC [3]–[5].

Numerous efforts have been applied to disclose the mechanisms that underlie the resistance of cancer cells to cisplatin-based chemotherapy or radiotherapy. The known mechanisms include defects in the DNA repair pathways, the promotion of cell survival signals, or decreased drug accumulation inside cancer cells [6]–[8]. Our recent efforts to explore the sensitivity factors for cisplatin treatment in NPC cells have revealed that asparagine synthetase, matrix metalloproteinase 19, and eIF3a confer cisplatin sensitivity to NPC cells [9], [10]. However, to our knowledge, no study has reported the promotion of cisplatin sensitivity in highly metastatic NPC cells.

Ras association family members (RASSFs) are a group of ten mammalian proteins that can directly bind to the Ras oncoprotein, and accumulating evidence has suggested that apoptosis promotion is a common characteristic of the RASSF family [11]–[15]. It has been reported that RASSF1A overcomes resistance to interferons [16], and RASSF1C is linked to DNA damage through activation of JNK signaling [17]. RASSF6 is similar to other RASSFs and is also linked to DNA repair [15], [18]. The relationship between RASSF6 and the DNA damage treatment response, however, has not been investigated.

In our previous study, we isolated a highly metastatic NPC cellular clone, S18, and a low metastatic clone, S26, from the parental NPC cell line CNE-2 [19]. It has been reported that S18 is more resistant to cisplatin treatment then its parental CNE-2 line and the low metastatic S26 clone [20]. Interestingly, our preliminary screening in the present study found that RASSF6 was the only member of the RASSF family that was remarkably upregulated in low metastatic S26 cells. We therefore hypothesized that RASSF6 could confer sensitivity to treatment in highly metastatic NPC cells. Our further explorations confirmed that restoring RASSF6 could induce treatment sensitivity in highly metastatic cells and that RASSF6 depletion could increase treatment resistance in low metastatic cells. The influence of RASSF6 in NPC cells partially depended on the regulation of apoptosis through the activation of JNK signaling because inhibition of the JNK pathway reduced the effect caused by RASSF6. Our study demonstrated that RASSF6 plays an important role in the cellular response to cisplatin and radiation treatment; therefore, RASSF6 could be used as a potential biomarker of NPC for predicting treatment response.

## Materials and Methods

### Ethics statement

The study was approved by the Ethics Committee of Sun-yat sen University Cancer Center. As all samples used in this study were anonymous and collected from patients for routine pathology use, no informed consent (written or verbal) was obtained for use of retrospective tissue samples from the patients in this study.

### Cell lines and culture

The human NPC cell lines/clones CNE-2, S26, and S18 (S26 and S18 were isolated from the parental line CNE-2 by limiting dilution method, as previously described [19]), and SUNE-1 and 5-8F (from the parental line SUNE-1 [21]) were maintained in DMEM (Invitrogen, CA) containing 10% fetal bovine serum (Invitrogen) in a humidified atmosphere with 5% CO_2_ at 37°C.

### Lentiviral transduction studies

A RASSF6 expression construct was generated by subcloning PCR-amplified, full-length human RASSF6 cDNA into the pCDH-CMV-MCS-EF1-RFP plasmid. Cells stably expressing either the RASSF6 short hairpin RNA (shRNA) or a scrambled, non-target shRNA were established using the LV3 plasmid according to the manufacturer's instructions. The targets of the RASSF6 shRNA-1 are 5′- GAACAAAGACGACTAAAGA-3′ and shRNA-3: 5′- GGAATTTGACGATCTCTAT-3′. Retroviral production and infection were performed as previously described [22], and stable cell lines were selected using 5 µg/ml puromycin for 7 days.

### Quantitative real-time PCR (qPCR)

Total RNA was extracted from cultured cells using TRIzol reagent (Invitrogen) according to the manufacturer's instructions and was reverse-transcribed using a cDNA Synthesis Kit (Takara, CA). Real-time qPCR was performed using a SYBR Green PCR Kit (Bio-Rad, CA), and expression of the target gene was normalized to endogenous levels of GAPDH. The relative mRNA levels were shown as the value of 2ΔCt. The PCR primer sequences that were used for amplification were as follows: GAPDH forward, 5′- AAGGTCATCCCTGAGCTGAA -3′; GAPDH reverse, 5′- TGACAAAGTGGTCGTTGAGG -3′; RASSF6 forward, 5′- GGGGGAATTTGACGATCTCT -3′; RASSF6 reverse, 5′- TAGAGCACTGGGGAGTCTGG -3′; RASSF1 forward 5′- GGACGGTTCTTACACAGGCT-3′; RASSF1 reverse 5′-CACCACCAAGAACTTTCGCA -3′; RASSF2 forward 5′-GCCAGAATTTACAGCTCCGG -3′; RASSF2 reverse 5′- GAACTTTGGGCACAGTCAGG-3′; RASSF3 forward 5′-ACCCACAGTTACCTCAGCAA -3′; RASSF3 reverse 5′-CACTTCCCCGACAGTGTTTG -3′; RASSF4 forward 5′-AGGCTGAGAGTTCCACAGAC -3′; RASSF4 reverse 5′- TCACATTGGTCACGGATCCA-3′; RASSF5 forward 5′- CTCTCCAGAAAGCACCCTCA-3′; and RASSF5 reverse 5′- CACTCAGTTTCATGCCCAGG-3′. The experiments were performed in triplicate.

### Cell viability assays

The cytotoxicities of cisplatin and radiation were determined using MTS assays (Cell Titer 96 Aqueous One Solution Cell Proliferation Assay solution; Promega, WI) and colony formation assays. For the MTS assay, cells were harvested and cultured in 96-well plates at a density of 2000 cells/well. Twenty four hours later, various concentration of cisplatin was added and the cells were incubated for 48 hours for drug treatment. For radiation treatment, the cells were treated with radiation at various dose 12 hours after plating the cells and then incubated for another 72 hours. The viable cells were then stained with MTS followed by determination of the OD490 using a microplate reader. For the colony formation assay, cells were plated at 500 cells per well in a 6-well plate (Corning, NY) for 24 hours and then radiated at various doses. Two weeks later, the cells were washed with phosphate buffered saline (PBS), fixed with methanol, and stained with crystal violet.

### Apoptosis assays

After treatment of the cells with cisplatin (48 hours) or radiation (72 hours), the floating and adherent cells were harvested, washed with PBS, and stained with Annexin-V-phycoerythrin (or Annexin-V-FITC) and 7-AAD (BD Biosciences, NJ) for 15 minutes at room temperature. The cells were then subjected to flow cytometry analysis (Beckman Coulter, Cytomics FC500, CA).

### Immunoblotting

Immunoblotting was performed according to the standard methods as described previously [22]. Briefly, cells were harvested, lysed on ice, and centrifuged (10,000 g for 30 min at 4°C) to remove debris. Supernatants were collected, and 50–100 g of total protein of each sample was subjected to SDS–PAGE/Western blotting. The blots were incubated with primary antibodies overnight at 4°C, followed by incubation with HRP-conjugated secondary antibodies (Promega) for 1 hour at room temperature. Finally, the signals were detected using an ECL system.

### Antibodies and chemical drugs

The antibodies that were used for Western blotting were as follows: RASSF6 (HPA037711, Sigma, CA); and human cleaved PARP (#6525), caspase 3 (#9662), SAPK/JNK (#9258), phospho-SAPK/JNK (#4668), c-Jun (#9165), phospho-c-Jun (Ser63, #9261), phospho-c-Jun (Ser73, #3270), ERK (#4780), phosphor-ERK1/2 (#5726), p38 (#8690), phosphor-p38 (#4511), β-actin (#4970), and GAPDH (#2118) were obtained from Cell Signaling Technology (BSN). SP600125 was purchased from Sigma and dissolved in 100% dimethyl sulfoxide (DMSO) at a concentration of 20 mM for storage.

### Statistical analyses

All statistical analyses were performed using the SPSS 17.0 statistical software package. Data from three independent experiments were presented as the mean values and standard deviations. The differences between two groups were evaluated using Student's t-test, and differences among three groups were evaluated using analysis of variance (ANOVA). *P* value <0.05 was considered to be statistically significant.

## Results

### Downregulation of RASSF6 in highly metastatic NPC cells correlates with resistance to treatment sensitivity

**Figure 1:**
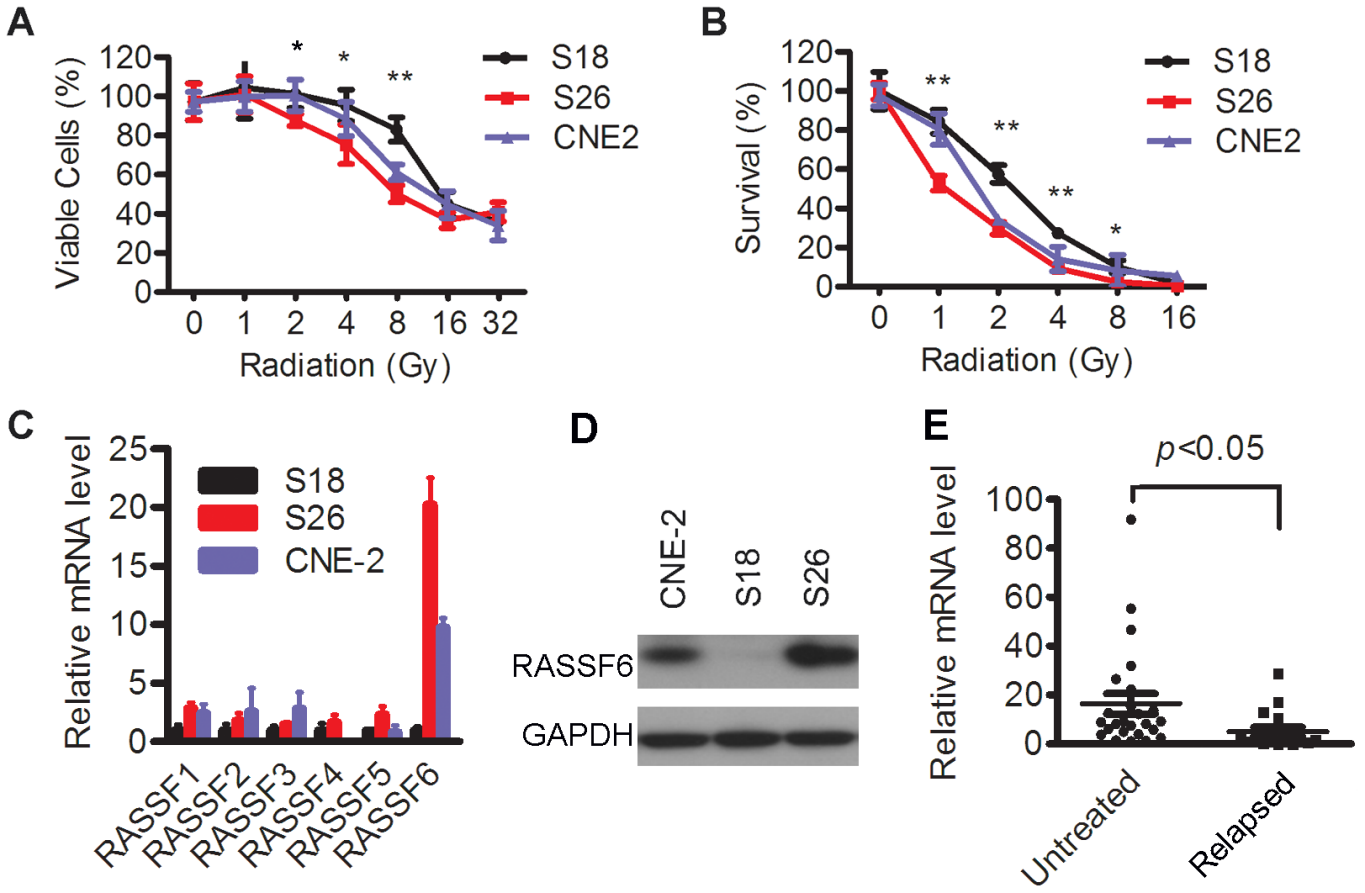
Characteristics of treatment sensitivity and RASSF6 expression in NPC cells and tissues. (**A, B**) CNE-2, S18, and S26 cells were treated with various doses of radiation. The viable cells were evaluated using an MTS assay (**A**); and the abilities of colony formation upon various radiation doses were compared. *P<0.05, **P<0.01, ANOVA. (**C**) mRNA expression of RASSF family members in CNE-2, S18, and S26 cells. (**D**) Western blot analysis of the RASSF6 protein level in CNE-2, S18, and S26 cells; GAPDH was used as a loading control. (**E**) RASSF6 mRNA expression levels in 18 relapsed NPC tissues (Relapsed) and 25 untreated NPC tissues (Untreated) were determined by qPCR assays. GAPDH and 18S were used as reference genes. *P*<0.05, student's t-test.

Highly metastatic S18 cells have been found to be more resistant to cisplatin treatment [20]. In the present study, we evaluated the radiotherapy sensitivities of S18, S26, and their parental line CNE-2. S18 cells displayed the lowest amount of irradiation-induced cell inhibition among the three cell lines, as shown using the MTS and colony formation assays (Figure 1A and 1B).

Then, the expression profile of the RASSF family in these three cell lines was analyzed. RASSF7∼10 belong to the N-terminal RASSFs and display different characteristics from the C-terminal RASSF1-6; therefore, we focused on RASSF1-6 in the present study. Compared with S18, the S26 line generally had higher expression levels of RASSF1-5. Interestingly, RASSF6 showed the greatest differential expression among the three cell lines (more than a 19-fold change between S18 and S26, Figure 1C). Western blotting also showed that the protein level was higher in S26 (Figure 1D). It is well known that the majority of NPCs are sensitive to radiotherapy and chemotherapy at the beginning, and only a small portion of NPCs becomes treatment-resistant manifesting as relapsed tumors. Therefore we compared 18 samples of relapsed NPC tissues, representing treatment resistance in some extents, versus 25 primary NPC tissues collected before treatment. RASSF6 mRNA expression in relapsed tissues was significantly lower than the expression in untreated tissues (Figure 1E, *p*<0.05).

### Over-expression of RASSF6 in highly metastatic NPC cells increases their sensitivity to cisplatin and radiation treatment

**Figure 2:**
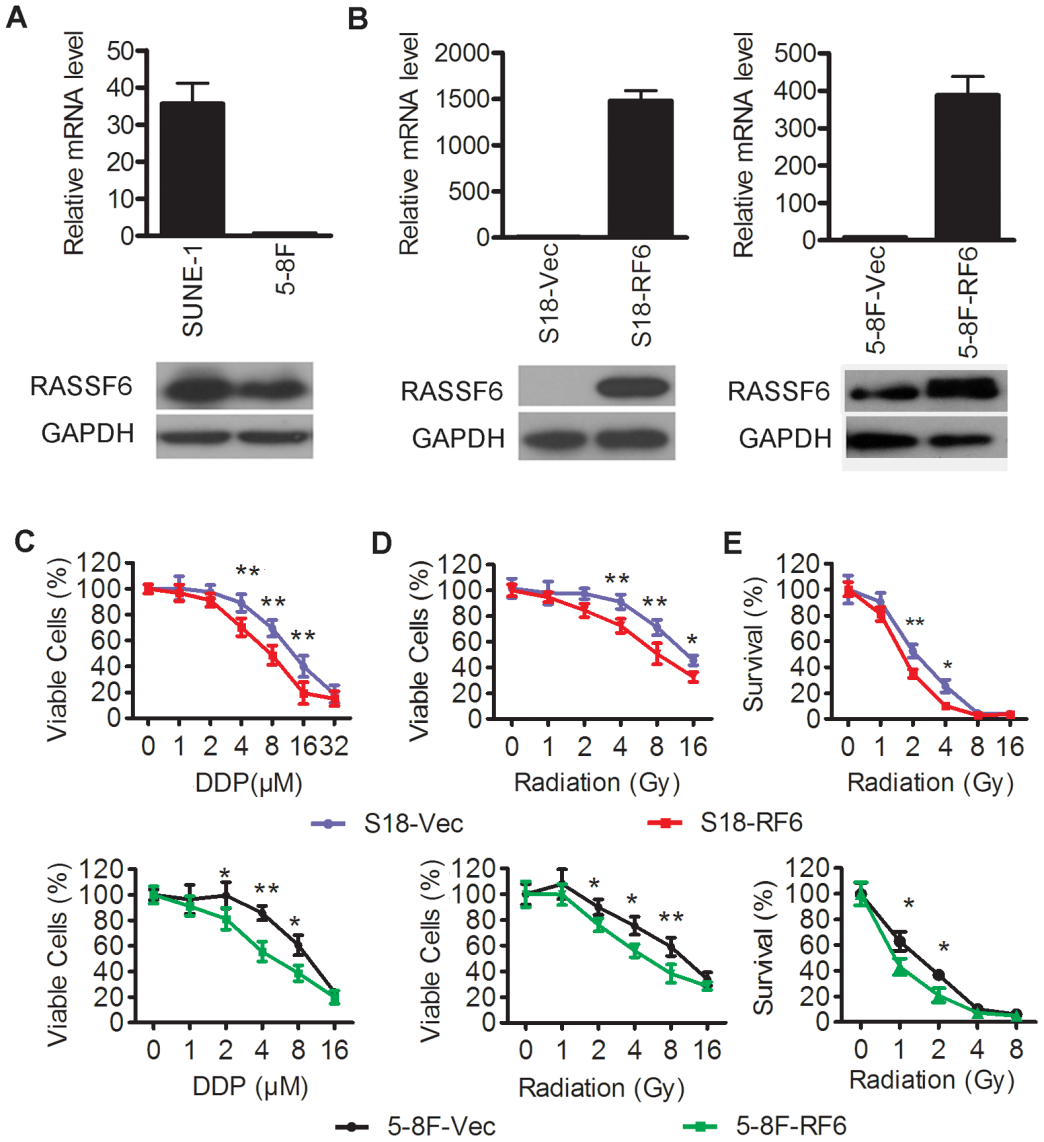
Overexpression of RASSF6 enhanced the sensitivities of highly metastatic NPC cells to cisplatin and radiotherapy. (**A**) mRNA (upper) and protein level (bottom) of RASSF6 in another highly metastatic clone, 5-8F, in contrast to its low metastatic parental line SUNE-1. (**B, C, D, E**) S18 and 5-8F cells with stable RASSF6 overexpression (RF6) or transfected with an empty vector control (Vec) were analyzed for: (**B**) RASSF6 mRNA (upper) and protein (lower) levels; (**C**) the response to various doses of cisplatin (DDP) treatment using MTS assays; (**D**) the response to various doses of radiation treatment using MTS assays; and (**E**) the ability for colony formation upon various radiation doses. *P<0.05, **P<0.01, Student's t test.

To assess whether RASSF6 alters the sensitivity of NPC cells toward cisplatin or radiation treatment and to determine the general role of RASSF6 in NPC cells, we compared another highly metastatic NPC cell line, 5-8F, to its parental, low metastatic cell line SUNE-1. 5-8F was more resistant to cisplatin treatment than SUNE-1(Figure S1A, IC50 of 9.94±1.86 µM in 5-8F cells *versus* IC50 of 5.92±1.09 µM in SUNE-1 cells). Compared to SUNE-1, 5-8F seemed to show no obvious different sensitivity to radiation (Figure S1B). 5-8F displayed lower RASSF6 expression compared with SUNE-1 (Figure 2A). Next, we effectively ectopically overexpressed RASSF6 in S18 and 5-8F cells (Figure 2B) and determined if the upregulation of RASSF6 would increase the sensitivity to cisplatin or radiation treatment. An MTS assay showed that the number of viable RASSF6-expressing S18 (Figure 2C upper, IC50 of 11.8±1.34 µM in S18 cells transfected with an empty vector *versus* IC50 of 6.37±0.68 µM in S18 cells with RASSF6 overexpression, *P*<0.01) and RASSF6-expressing 5-8F cells (Figure 2C bottom, IC50 of 9.85±1.04 µM in 5-8F cells transfected with an empty vector *versus* IC50 of 5.6±0.42 µM in the 5-8F cells with RASSF6 overexpression, *P*<0.05) decreased after treatment with cisplatin. Then, the cells were treated with various doses of radiation. The same dose of radiation led to a higher rate of cell death in RASSF6-transfected S18 or 5-8F cells in contrast to their respective control cells (Figure 2D). To rule out the effect of early-accelerated repopulation during radiotherapy, we tested for colony formation ability in these cells. Again, we found that RASSF6-transfected S18 or 5-8F cells formed fewer colonies compared to the control cells, even at low doses of radiation therapy (Figure 2E). Together, these data proved that overexpression of RASSF6 in highly metastatic NPC cells could significantly sensitize the cells to cisplatin treatment and radiotherapy.

### Knockdown of RASSF6 in low metastatic NPC cells reduces their sensitivity to cisplatin and radiation treatment

**Figure 3:**
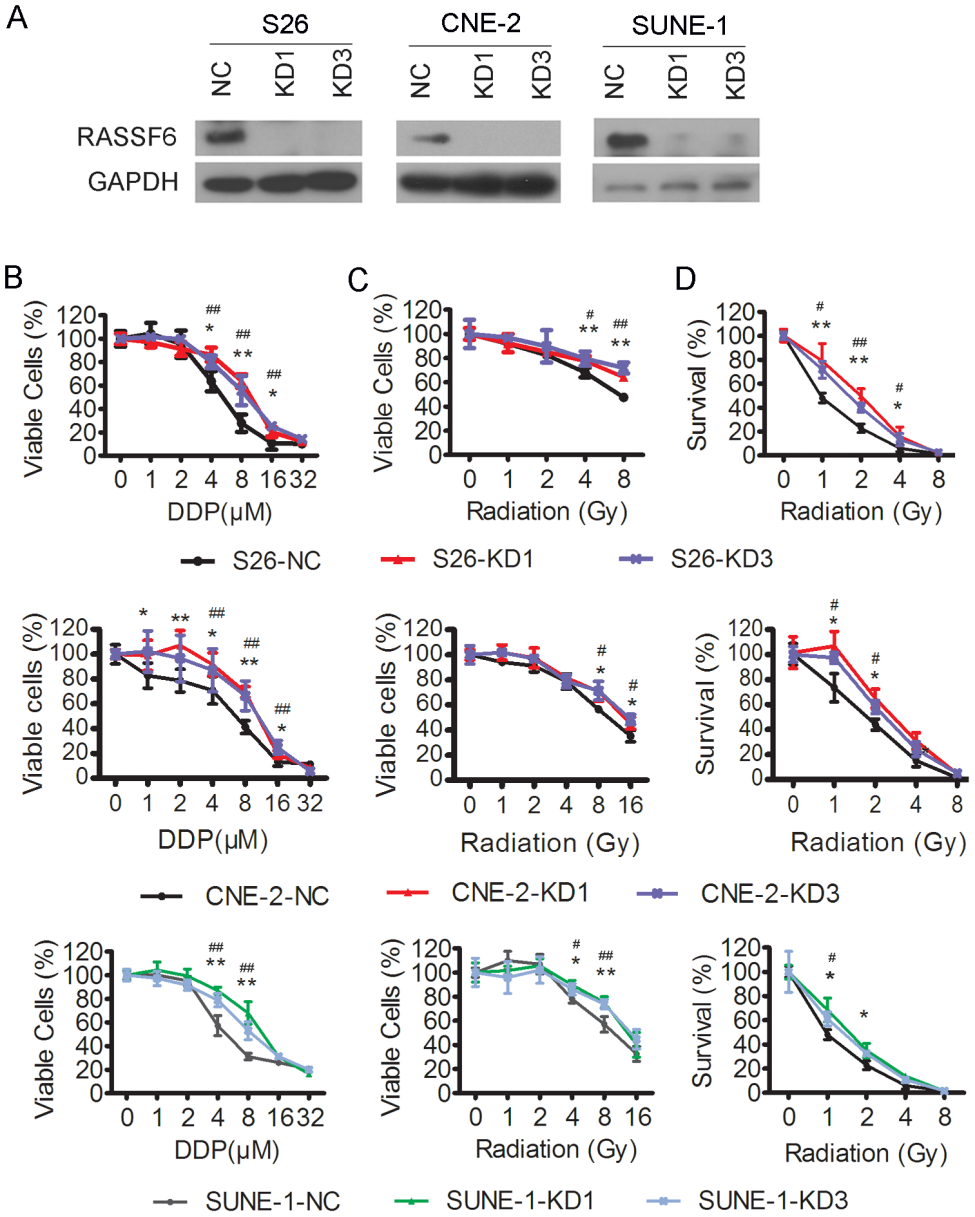
Depletion of RASSF6 increases the resistance of low metastatic NPC cells to cisplatin and radiotherapy. S26,CNE-2 and SUNE-1 cells were stably transfected with two different RASSF6 shRNAs (KD1, KD3) or a negative control sh-RNA (NC), followed by (**A**) Western blot analysis of RASSF6 expression, with GAPDH used as a loading control; (**B**) an MTS assay of the cellular response to various doses of cisplatin (DDP); (**C**) an MTS assay of the cellular response to various doses of radiation treatment; and (**D**) the abilities of colony formation upon various doses of radiation treatment. *P<0.05, **P<0.01 for KD1 cells compared with NC cells, # *P*<0.05, ##*P*<0.01 for KD3 cells compared with NC cells, Student's t test.

We then performed the reverse experiment by knocking down RASSF6 in low metastatic S26, CNE-2 and SUNE-1 cells and determined whether the reduction in RASSF6 expression would decrease the cellular sensitivity to cisplatin or radiation treatment. As shown in Figure 3A, RASSF6 could be effectively knocked down in S26, CNE-2 and SUNE-1 cells by sh-RNAs against RASSF6. S26 cells with RASSF6 knocked down were significantly more resistant to cisplatin than those transfected with the negative control vector (Figure 3B upper, IC50 of 4.8±0.39 µM in S26 transfected with vector (NC) *versus* IC50 of 9.1±0.75 µM and 8.6±0.89 µM in S26 cells transfected with RASSF6 shRNA-1 (KD1) and shRNA-3 (KD3), respectively, *P*<0.05 for both comparisons). Consistent results were observed in CNE-2 cells (Figure 3B middle, IC50 of 5.88±0.54 µM in CNE-2 cells transfected with vector (NC) *versus* IC50 of 10.4±0.75 µM and 11.62±1.7 µM in CNE-2 cells transfected with RASSF6 shRNA 1 (KD1) and shRNA3 (KD3), respectively, *P*<0.05 for both comparisons) and SUNE-1 cells upon cisplatin treatment (Figure 3C bottom, IC50 of 6.72±0.87 µM in SUNE-1 cells transfected with vector (NC) *versus* IC50 of 10.82±1.45 µM and 9.1±1.13 µM in SUNE-1 cells transfected with RASSF6 shRNA 1 (KD1) and shRNA3 (KD3), respectively, *P*<0.05 for both comparisons). After irradiation, more viable cells were detected in the S26,CNE-2 and SUNE-1 cells with RASSF6 stably knocked down (Figure 3C). The ability of the low metastatic cells to form colonies was also enhanced after knocking down RASSF6 (Figure 3D). These data further confirmed that RASSF6 played a role in the cellular sensitivity to cisplatin and radiation treatment.

### RASSF6 regulates cisplatin/radiation-induced apoptosis

**Figure 4:**
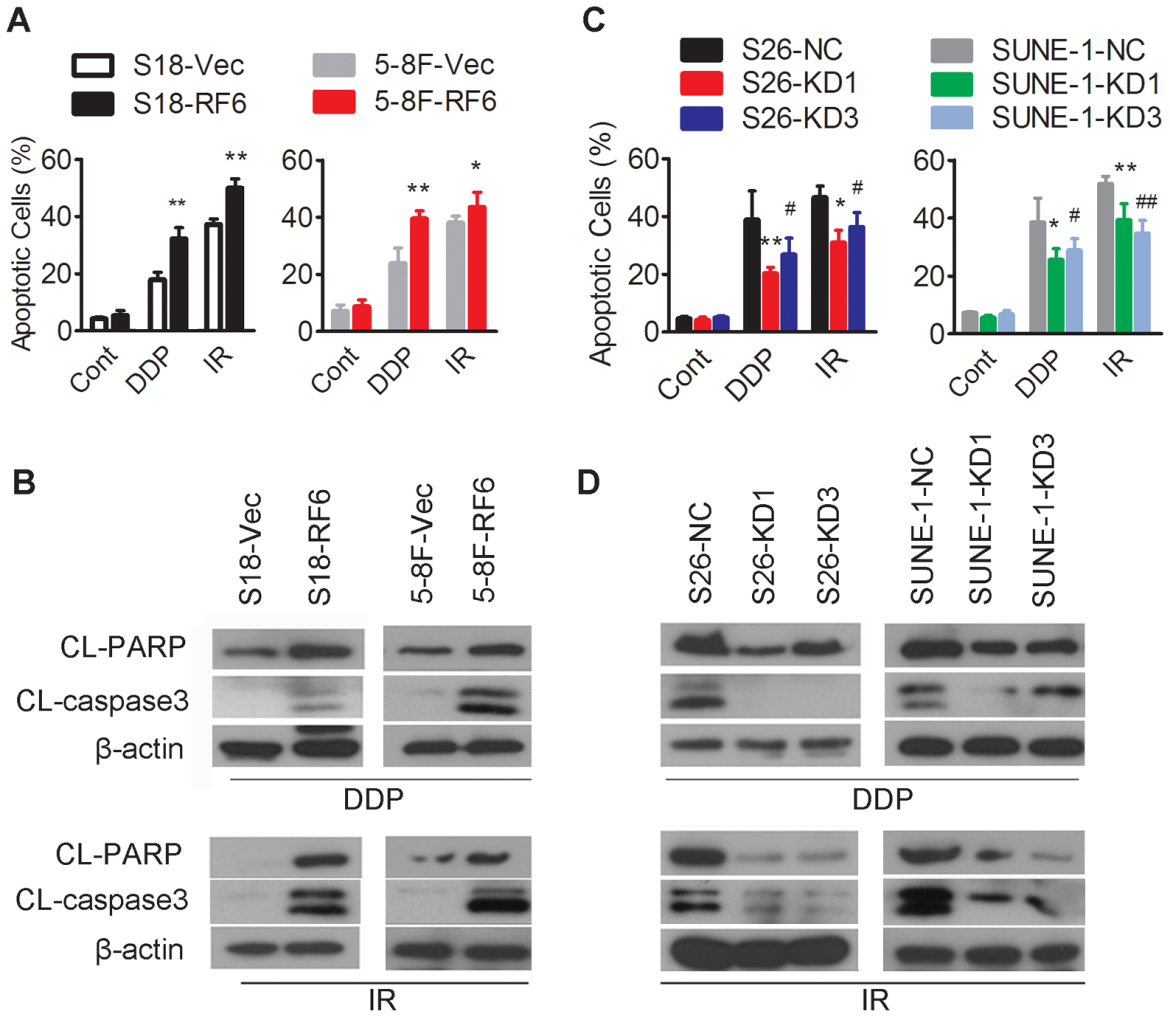
Upon cisplatin or radiation treatment, RASSF6 overexpression increased apoptosis, and RASSF6 depletion reduced apoptosis. (**A, B**) S18 and 5-8F cells with stable RASSF6 overexpression (RF6) or transfected with an empty vector control (Vec) were treated with the indicated doses of cisplatin (DDP, 6 µM for S18 and 8 µM for 5-8F cells), radiation (IR, 8 Gy for S18 and 5-8F cells) or not treated (Cont). Cells were collected for (**A**) flow cytometry analysis of apoptosis, *P<0.05, **P<0.01, Student's t test, and (**B**) Western blotting (upper panel for cisplatin treatment, lower panel for radiation treatment) for apoptosis-related proteins, including cleaved PARP and caspase 3, and β-actin as a loading control. (**C, D**) S26 and SUNE-1 cells stably transfected with two RASSF6 shRNAs (KD1, KD3) or with the negative control sh-RNA (NC) were treated with the indicated doses of cisplatin (DDP, 6 µM for S26 and 8 µM for SUNE-1) or radiation (IR, 8 Gy for S26 and SUNE-1) or no treated (Cont). Cells were collected for (**C**) flow cytometry analysis of apoptosis (*P<0.05, **P<0.01 for KD1 cells compared with NC cells, # *P*<0.05, ## *P*<0.01 for KD3 cells compared with NC cells, Student's t test); and (**D**) Western blotting (upper panel for cisplatin treatment, lower panel for radiation treatment) for apoptosis-related proteins, including cleaved PARP and caspase 3, and β-actin as a loading control.

Cisplatin and radiation treatment are known to exert their cytotoxicity by inducing apoptosis. RASSF6 could induce apoptosis in various cells when exposed to DNA damage treatment [15], [23]. Against this background, we tested whether RASSF6 mediates the treatment response via apoptosis by comparing Annexin V/7-AAD staining and the expression of cleaved-PARP or caspase 3, both of which serve as markers for cells undergoing apoptosis. S18 and 5-8F cells overexpressing RASSF6 had significantly higher apoptosis and elevated levels of cleaved PARP and caspase-3 when exposed to cisplatin or radiation treatment than cells transfected with the empty vector (Fig. 4A and 4B, Figure S2). In contrast, knockdown of RASSF6 in S26 or SUNE-1 cells reduced cisplatin- or radiation-induced apoptosis and the expression level of cleaved PARP and caspase-3 (Fig. 4C and 4D, Figure S3).

### RASSF6 promotes cisplatin/radiation sensitivity by activating JNK signaling

**Figure 5:**
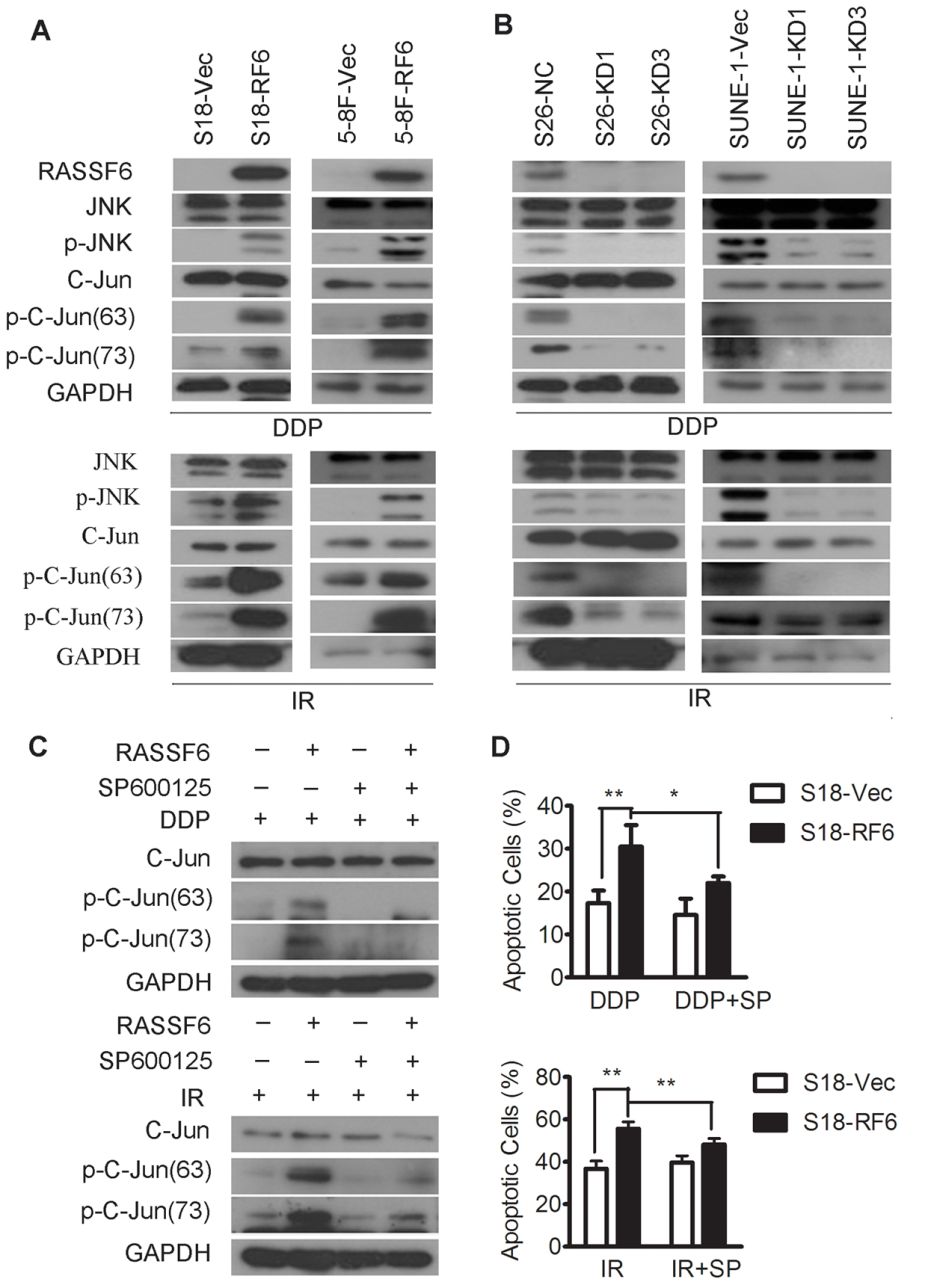
JNK signaling was activated by RASSF6 during the cellular response to cisplatin and radiation treatment. (**A**) S18 (left) and 5-8F (right) cells stably overexpressing RASSF6 (RF6) or transfected with an empty vector control (Vec) were treated with the indicated doses of cisplatin (upper panel, DDP, 6 µM for S18 and 8 µM for 5-8F cells) or radiation (lower panel, IR, 8 Gy for S18 and 5-8F cells). Protein was collected for Western blot analysis to evaluate the expression of RASSF6, phosphorylated JNK, total JNK, phosphorylated C-jun (ser63), phosphorylated C-jun (ser73), total C-jun, and GAPDH as a loading control. (**B**) S26 (left) and SUNE-1 (right) cells stably transfected with two RASSF6 shRNAs (KD1, KD3) or with a negative control sh-RNA (NC) were treated with the indicated doses of cisplatin (upper panel, DDP, 6 µM for S26 and 8 µM for SUNE-1 cells) or radiation (lower panel, IR, 8 Gy for S26 and SUNE-1 cells). Protein was collected for Western blot analysis of the expression of RASSF6, phosphorylated JNK, total JNK, phosphorylated C-jun (ser63), phosphorylated C-jun (ser73), total C-jun, and GAPDH as a loading control. (**C, D**) S18 cells stably overexpressing RASSF6 (RF6) or transfected with an empty vector control (Vec) were treated with cisplatin (DDP, 6 µM) or radiation (IR, 8 Gy) in the presence or absence (0.1% DMSO was used as a control) of the JNK inhibitor SP600125 (8 µM). The cells were then collected for (**C**) Western blot analysis of the expression of phosphorylated C-jun (ser63), phosphorylated C-jun (ser73), and total C-jun, with GAPDH as a loading control; and (**D**) flow cytometry analysis of apoptosis after exposure to cisplatin or radiation treatment, *P<0.05, **P<0.01, Student's t test.

We then explored the underlying mechanism of RASSF6-promoted apoptosis. It has been reported that the RAS- MAPK signaling pathways play an important role in cellular regulation, including apoptosis caused by RASSF members. Therefore, we tested the MAPK activity in NPC cells overexpressing or depleted of RASSF6. Overexpression of RASSF6 in highly metastatic NPC cells specifically enhanced the protein level of phosphorylated JNK and C-jun (Figure 5A), but not of p38 kinase or ERK (Figure S4), when exposed to cisplatin or radiation treatment. On the other hand, phosphorylation of JNK and C-jun were inhibited in RASSF6-depleted NPC cells (Figure 5B). To confirm that the involvement of JNK signaling is essential for the RASSF6-promoted sensitivity to chemotherapy and radiotherapy in NPC cells, we applied the JNK inhibitor SP600125 to cells exposed to cisplatin or radiation treatment (Figure 5C). As expected, pharmacologic inhibition of JNK clearly suppressed the phosphorylation of c-Jun that was caused by RASSF6 and partially blocked RASSF6-promoted apoptosis (Figure 5D, Figure S5). Taken together, these results suggested that the RASSF6-promoted sensitivity to chemotherapy and radiotherapy depended on the activation of JNK signaling in NPC cells.

## Discussion

Metastatic cancers are usually resistant to conventional chemotherapy and/or radiation therapy [24]–[26]. Successful metastasis involves the interaction of cancer cells with a compatible, organ-specific environment. The use of organ-derived molecules (e.g., P-gp, fibroblast growth factor, hepatocyte growth factor) is one important mechanism for cancer cell metastasis, and these mechanisms influence response of metastatic tumor cells to chemo-/radio-therapy [27]. Some believe that the metastatic cancer cells themselves are responsible for treatment resistance by gaining stemness characteristics within metastatic lesions [28]–[30]. The development of treatment resistance in a highly metastatic tumor is a complex process, and it is unrealistic to expect that any single mechanism will uncover the truth [31]. More studies are needed to investigate the conditions by which treatment resistance is linked or not linked to metastasis and the signals or factors that may be relevant.

RASSF6 has been found to be tightly associated with tumorigenesis and functions as a tumor suppressor. RASSF6 harbors Ras-association (RA) domains in its C-terminal region that are conserved in the primary structure [18]. RASSF6 is involved in apoptosis in various cells with treatment with pro-apoptotic agents by triggering caspase-dependent and caspase-independent pathways or a Hippo-independent pathway [15], [32]. RASSF6 also increases DNA repair in ultraviolet- and VP-16-treated cells [33]. We report here, for the first time, that the downregulation of RASSF6 in highly metastatic NPC cells conferred resistance to cisplatin and radiation treatment.

RAS conveys growth/stress stimuli from the cell surface to the inner cell through RAS- MAPK signaling, which mainly includes the stress-activated protein kinase/c-Jun-N-terminal kinase (JNK), p38 kinase, and extracellular signal-regulated kinase (ERK) pathways. The Ras-association domain (RA), which is a characteristic feature of the RASSF family, confers a direct binding ability to RAS. Consequently, RASSF members most likely affect the activity of MAPK pathways. In fact, some other RASSF members have been found to induce apoptosis through the inactivation of ERK [34], [35] and the activation of SAPK/JNK [17]. In the present study, we observed that cisplatin or radiation treatment enhanced JNK activation in RASSF6-expressing cells. The importance of JNK activation in the cell response to anti-cancer drug and resistance development has been investigated in recent years [7]. DNA damage treatment could activate a robust apoptotic response that involves activation of the JNK pathway, but it failed to elicit JNK activation in treatment-resistant cells. Furthermore, inhibition of JNK activation partially restored the sensitivity of cancer cells to cisplatin [36]–[38]. Consistent with previous reports, specific inhibition of the JNK pathway in our study significantly reduced RASSF6-promoted apoptosis, which further confirmed a role for JNK signaling in RASSF6-mediated regulation of the treatment response.

Taken together, our results demonstrate that downregulation of RASSF6 was responsible for the treatment resistance of highly metastatic NPC cells and that over-expression of RASSF6 and the subsequent activation of JNK signaling conferred treatment sensitivity to NPC cells. RASSF6 could be a valuable molecule for the reversal of treatment resistance in NPC, especially for metastatic lesions.

## Supporting Information

Figure S1 The response of highly metastatic 5-8F cells and low metastatic SUNE-1 cells to cisplatin and radiotherapy. 5-8F and SUNE-1cells were treated with various doses of cisplatin (**A**) or radiation (**B**). The viable cells were evaluated using an MTS assay. *P<0.05, **P<0.01, student's t-test.
(TIF)

Figure S2 Representative images of Annexin-V and 7-AAD double staining of high-metastatic NPC cells upon cisplatin and radiation treatment. S18 (**A**) and 5-8F cells (**B**) stably overexpressing RASSF6 (RF6) or transfected with an empty vector (Vec) were untreated (Control) or treated with the indicated doses of cisplatin (DDP, 6 µM for S18 and 8 µM for 5-8F cells) or radiation (IR, 8 Gy for S18 and 5-8F cells). The cells were then collected for flow cytometry analysis of apoptosis using Annexin-V and 7-AAD double staining.
(TIF)

Figure S3 Representative images of Annexin-V and 7-AAD double staining of low-metastatic NPC cells upon cisplatin and radiation treatment. S26 (**A**) and SUNE-1 cells (**B**) stably transfected with two RASSF6 shRNAs (KD1, KD3) or the negative control sh-RNA (NC) were untreated (Control) or treated with the indicated doses of cisplatin (DDP, 6 µM for S26 and 8 µM for SUNE-1 cells) or radiation (IR, 8 Gy for S26 and SUNE-1 cells). Apoptotic cells were evaluated using Annexin-V and 7-AAD double staining and flow cytometry.
(TIF)

Figure S4 RASSF6 regulates the response of NPC cells to cisplatin and radiation treatment independent of ERK or P38 signaling. S18 cells stably overexpressing RASSF6 (RF6) or an empty vector (Vec) and S26 cells stably transfected with two RASSF6 shRNAs (KD1, KD3) or the negative control sh-RNA (NC) were treated with cisplatin (DDP, 6 µM) or radiation (IR, 8 Gy). The whole cell lysate was collected for immunoblotting for phosphorylated ERK, total ERK, phosphorylated p38, and total p38 proteins. β-actin was used as a loading control.
(TIF)

Figure S5 Inhibition of JNK signaling partially blocked the RASSF6-induced apoptosis in highly metastatic NPC cells. (**A**) S18 cells stably overexpressing RASSF6 (RF6) or an empty vector (Vec) were treated with cisplatin (DDP, 6 µM) or radiation (IR, 8 Gy) in the presence or absence (0.1% DMSO was used as control) of the JNK inhibitor SP600125 (8 µM). Apoptosis was determined using flow cytometry. (**B, C**) 5-8F cells stably overexpressing RASSF6 (RF6) or transfected with an empty vector control (Vec) were treated with cisplatin (DDP, 8 µM) or radiation (IR, 8 Gy) in the presence or absence (0.1% DMSO was used as a control) of the JNK inhibitor SP600125. The cells were collected for apoptotic analysis using flow cytometry (**B**) and quantification of the apoptotic index from the triplicate experiments is shown in (**C**), *p<0.05, **p<0.01, Student's t test.
(TIF)
