# Supplementary figures and images for: Downregulation of Ras Association Domain Family Member 6 (RASSF6) Underlies the Treatment Resistance of Highly Metastatic Nasopharyngeal Carcinoma Cells
Source: PLoS One. 2014 Jul 16;9(7):e100843. doi: 10.1371/journal.pone.0100843 (PMC4100732; doi:10.1371/journal.pone.0100843)

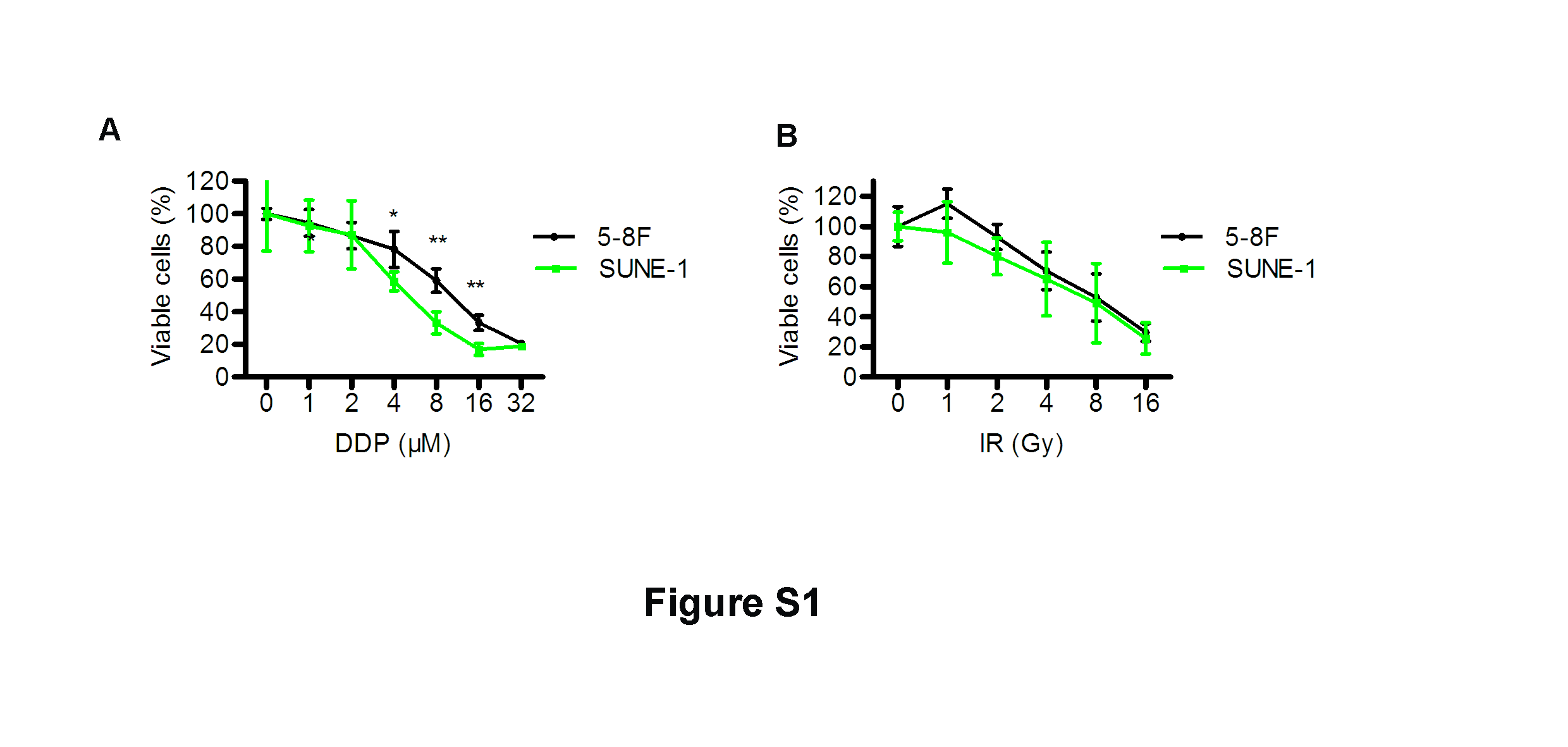

Supplement: Figure S1 — The response of highly metastatic 5-8F cells and low metastatic SUNE-1 cells to cisplatin and radiotherapy. 5-8F and SUNE-1cells were treated with various doses of cisplatin (A) or radiation (B). The viable cells were evaluated using an MTS assay. *P<0.05, **P<0.01, student's t-test. (TIF) [file pone.0100843.s001.tif]

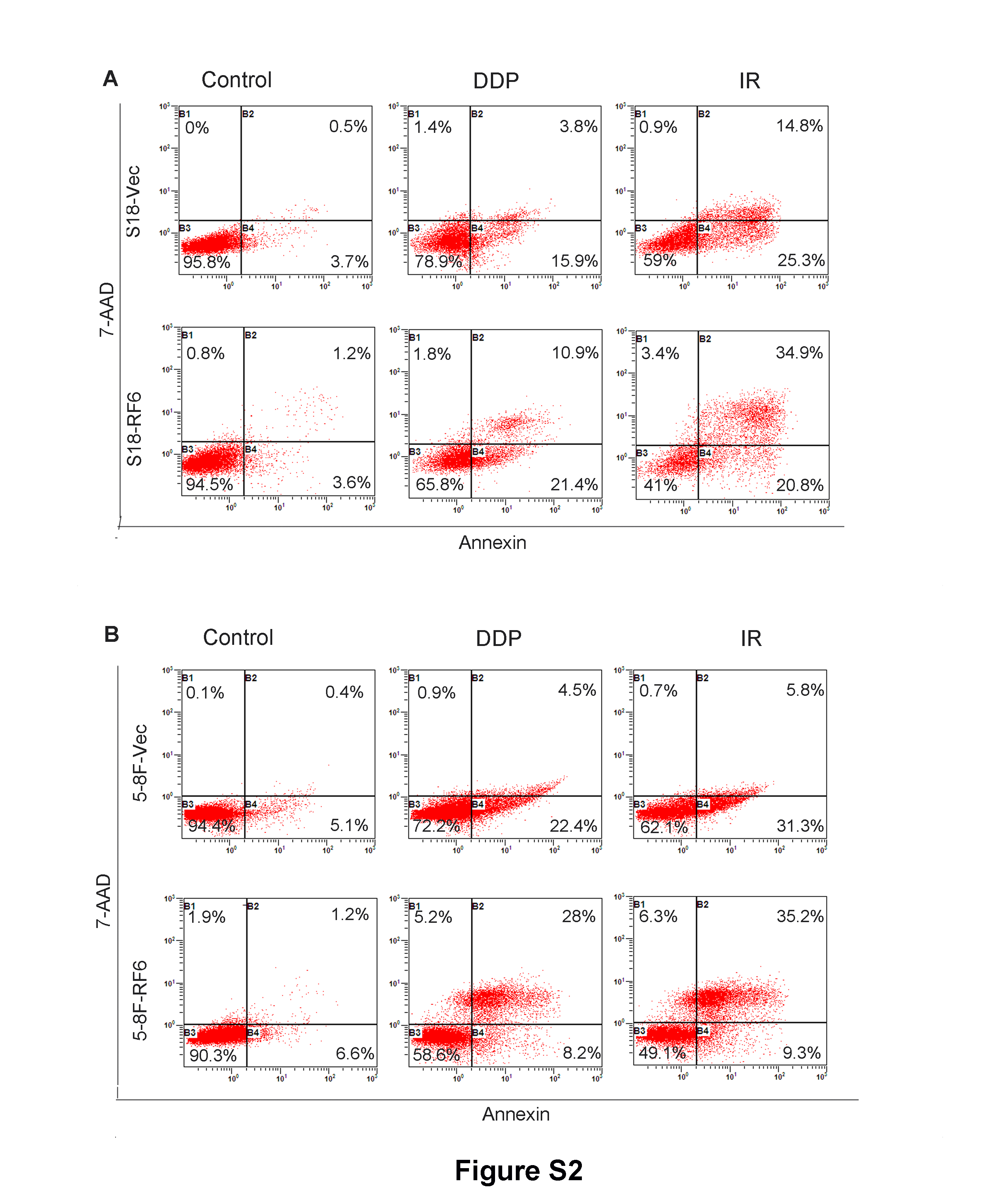

Supplement: Figure S2 — Representative images of Annexin-V and 7-AAD double staining of high-metastatic NPC cells upon cisplatin and radiation treatment. S18 (A) and 5-8F cells (B) stably overexpressing RASSF6 (RF6) or transfected with an empty vector (Vec) were untreated (Control) or treated with the indicated doses of cisplatin (DDP, 6 µM for S18 and 8 µM for 5-8F cells) or radiation (IR, 8 Gy for S18 and 5-8F cells). The cells were then collected for flow cytometry analysis of apoptosis using Annexin-V and 7-AAD double staining. (TIF) [file pone.0100843.s002.tif]

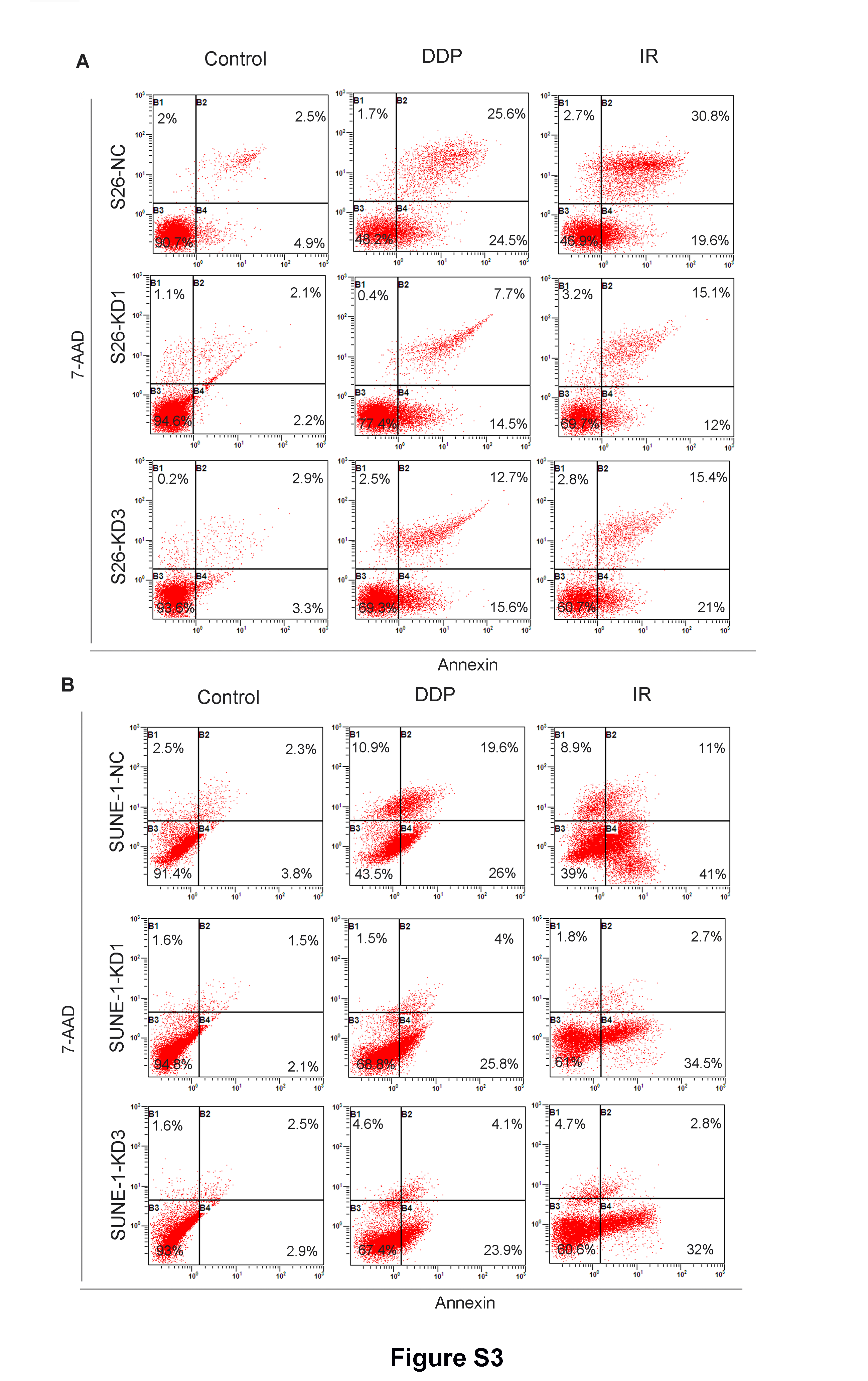

Supplement: Figure S3 — Representative images of Annexin-V and 7-AAD double staining of low-metastatic NPC cells upon cisplatin and radiation treatment. S26 (A) and SUNE-1 cells (B) stably transfected with two RASSF6 shRNAs (KD1, KD3) or the negative control sh-RNA (NC) were untreated (Control) or treated with the indicated doses of cisplatin (DDP, 6 µM for S26 and 8 µM for SUNE-1 cells) or radiation (IR, 8 Gy for S26 and SUNE-1 cells). Apoptotic cells were evaluated using Annexin-V and 7-AAD double staining and flow cytometry. (TIF) [file pone.0100843.s003.tif]

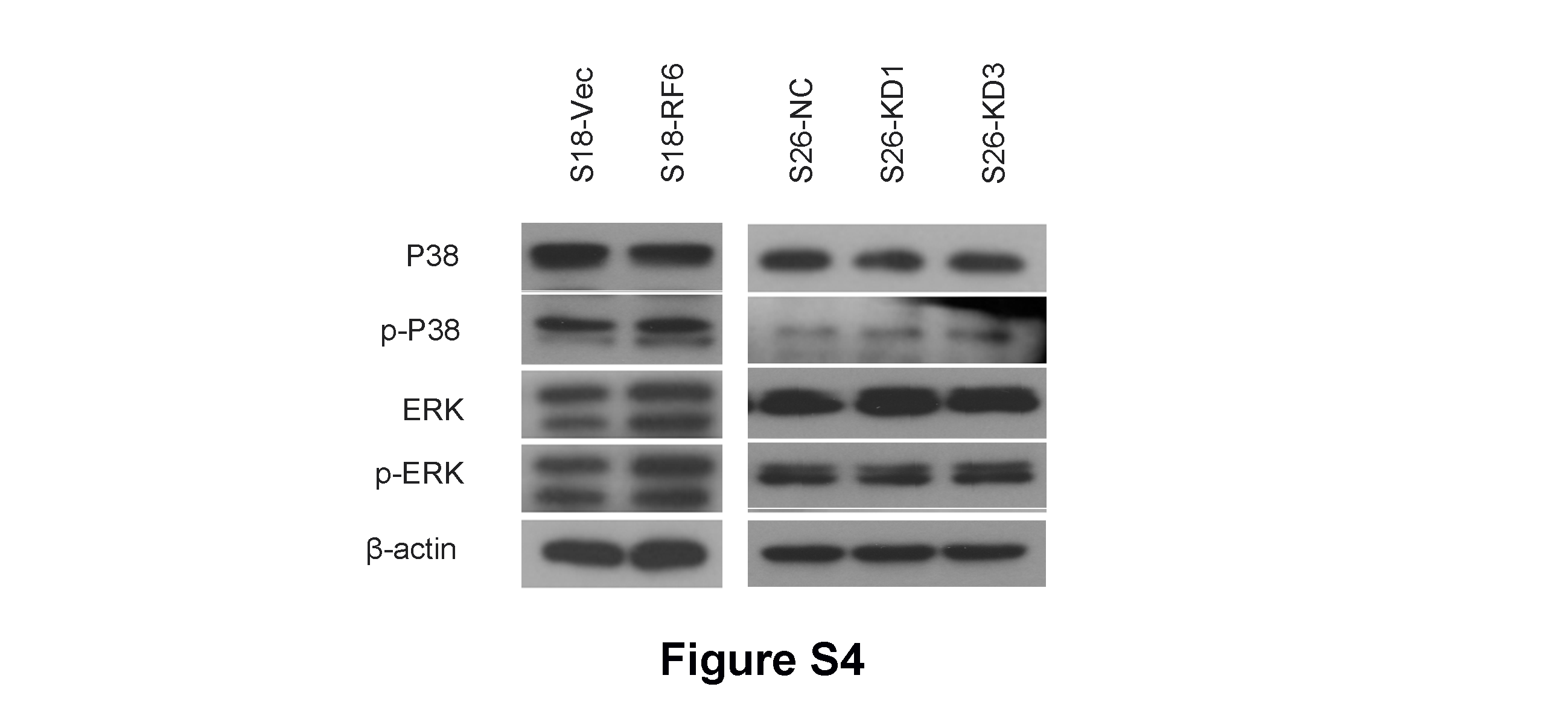

Supplement: Figure S4 — RASSF6 regulates the response of NPC cells to cisplatin and radiation treatment independent of ERK or P38 signaling. S18 cells stably overexpressing RASSF6 (RF6) or an empty vector (Vec) and S26 cells stably transfected with two RASSF6 shRNAs (KD1, KD3) or the negative control sh-RNA (NC) were treated with cisplatin (DDP, 6 µM) or radiation (IR, 8 Gy). The whole cell lysate was collected for immunoblotting for phosphorylated ERK, total ERK, phosphorylated p38, and total p38 proteins. β-actin was used as a loading control. (TIF) [file pone.0100843.s004.tif]

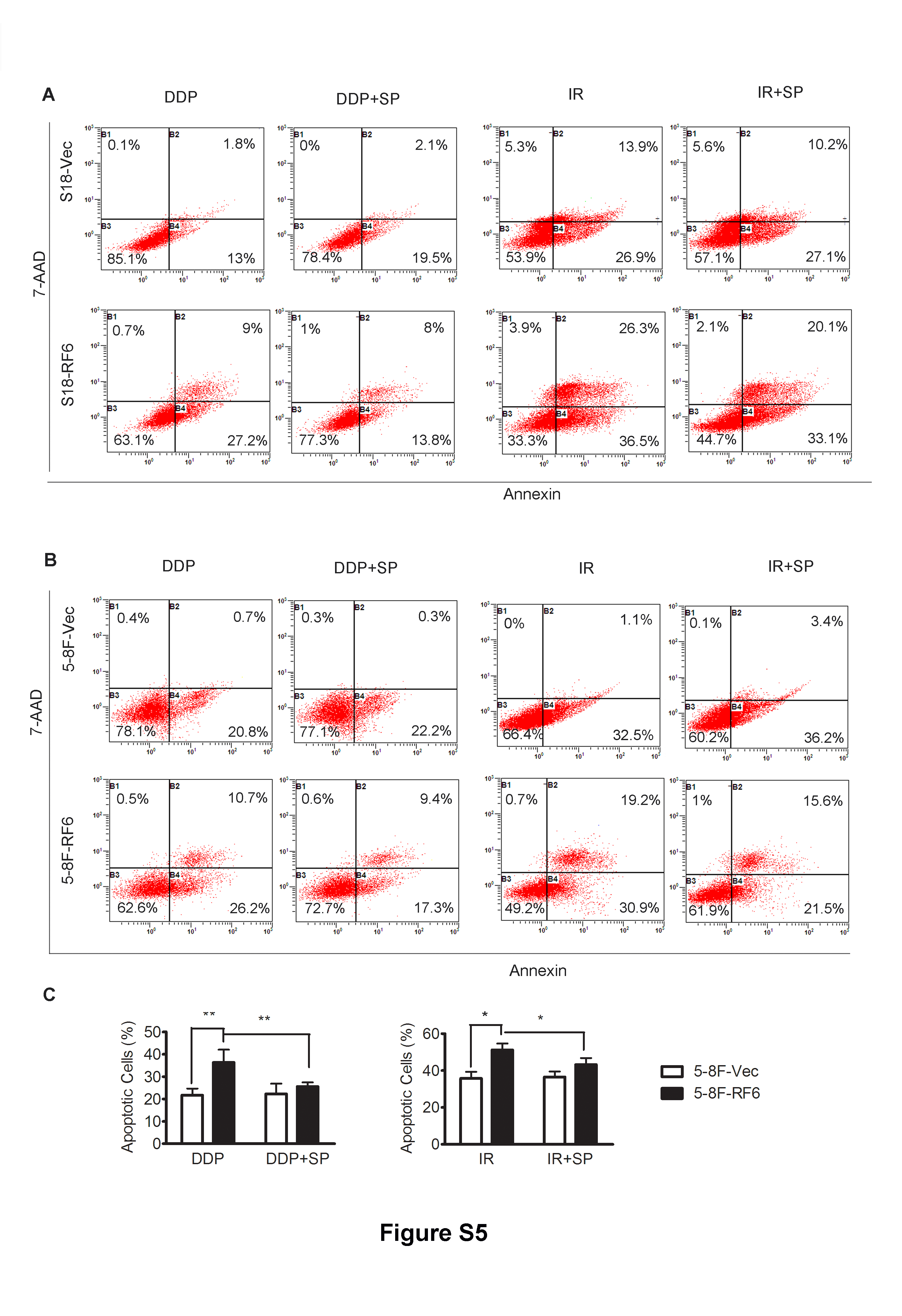

Supplement: Figure S5 — Inhibition of JNK signaling partially blocked the RASSF6-induced apoptosis in highly metastatic NPC cells. (A) S18 cells stably overexpressing RASSF6 (RF6) or an empty vector (Vec) were treated with cisplatin (DDP, 6 µM) or radiation (IR, 8 Gy) in the presence or absence (0.1% DMSO was used as control) of the JNK inhibitor SP600125 (8 µM). Apoptosis was determined using flow cytometry. (B, C) 5-8F cells stably overexpressing RASSF6 (RF6) or transfected with an empty vector control (Vec) were treated with cisplatin (DDP, 8 µM) or radiation (IR, 8 Gy) in the presence or absence (0.1% DMSO was used as a control) of the JNK inhibitor SP600125. The cells were collected for apoptotic analysis using flow cytometry (B) and quantification of the apoptotic index from the triplicate experiments is shown in (C), *p<0.05, **p<0.01, Student's t test. (TIF) [file pone.0100843.s005.tif]
